# Anti-tumor Effects of the eIF4A Inhibitor Didesmethylrocaglamide and Its Derivatives in Human and Canine Osteosarcomas

**DOI:** 10.21203/rs.3.rs-4494024/v1

**Published:** 2024-06-14

**Authors:** Janet Oblinger, Jack Wang, Georgia Wetherell, Garima Agarwal, Tyler Wilson, Nicole Benson, Joelle Fenger, James Fuchs, A Douglas Kinghorn, Long Chang

**Affiliations:** Abigail Wexner Research Institute at Nationwide Children’s Hospital; Abigail Wexner Research Institute at Nationwide Children’s Hospital; Abigail Wexner Research Institute at Nationwide Children’s Hospital; The Ohio State University; The Ohio State University; The Ohio State University; The Ohio State University; The Ohio State University; The Ohio State University; Abigail Wexner Research Institute at Nationwide Children’s Hospital

**Keywords:** Osteosarcoma, didesmethylrocaglamide (DDR), rocaglamide (Roc), eIF4A inhibitor, p38 stress-activated protein kinase, RhoB

## Abstract

Inhibition of translation initiation using eIF4A inhibitors like (−)-didesmethylrocaglamide [(−)-DDR] and (−)-rocaglamide [(−)-Roc] is a potential cancer treatment strategy as they simultaneously diminish multiple oncogenic drivers. We showed that human and dog osteosarcoma cells expressed high levels of eIF4A1/2, particularly eIF4A2. Genetic depletion of eIF4A1 and/or 2 slowed osteosarcoma cell growth. To advance preclinical development of eIF4A inhibitors, we demonstrated the importance of (−)-chirality in DDR for growth-inhibitory activity. Bromination of DDR at carbon-5 abolished growth-inhibitory activity, while acetylating DDR at carbon-1 was tolerated. Like DDR and Roc, DDR-acetate increased the γH2A.X levels and induced G_2_/M arrest and apoptosis. Consistent with translation inhibition, these rocaglates decreased the levels of several mitogenic kinases, the STAT3 transcription factor, and the stress-activated protein kinase p38. However, phosphorylated p38 was greatly enhanced in treated cells, suggesting activation of stress response pathways. RNA sequencing identified *RHOB* as a top upregulated gene in both DDR- and Roc-treated osteosarcoma cells, but the Rho inhibitor Rhosin did not enhance the growth-inhibitory activity of (−)-DDR or (−)-Roc. Nonetheless, these rocaglates potently suppressed tumor growth in a canine osteosarcoma patient-derived xenograft model. These results suggest that these eIF4A inhibitors can be leveraged to treat both human and dog osteosarcomas.

## Introduction

Osteosarcomas are highly aggressive bone malignancies typically found in patients younger than 20 years old^1^. These cancers tend to develop in the long bones of the axial skeleton. For decades, the standard-of-care has been extensive surgery, possibly including amputation of the affected limb, along with multi-agent cytotoxic chemotherapy. Although the cure rate for localized disease is ~ 70%, this cancer recurs in about one-third of patients. In addition, metastatic disease frequently occurs and usually targets the lungs. Patients with recurrent or metastatic disease have a dismal prognosis with < 20% survival beyond three years. Identification of a more effective medical therapy for osteosarcomas is direly needed.

While osteosarcomas exhibit low mutational burden^2^, they frequently have highly individualized copy-number alterations and structural rearrangements^3^. Due to complex genomic aberrations, osteosarcomas show dysregulation of multiple mitogenic signaling pathways, which are potential therapeutic targets^4^. However, clinical trials of therapies inhibiting individual targets have been disappointing, showing at best partial efficacy. Also, osteosarcomas harbor a dynamic tumor microenvironment (TME), which plays an important role in driving tumor growth and promoting treatment resistance^5^. These findings highlight the likely need to block simultaneously multiple signaling pathways to effectively treat these aggressive bone cancers.

Although osteosarcomas are the most common primary bone malignancies, they are relatively rare in humans with an incidence of less than 1000 cases per year in the United States. In contrast, osteosarcomas are more frequently found in dogs, particularly in certain large breeds^6^. The incidence of osteosarcomas in dogs is over 10-fold higher than in humans, with > 10,000 new canine patients each year. Remarkably, dog osteosarcomas closely recapitulate the human disease in many key features, including histopathology, clinical course, molecular genetics, and deregulated signaling pathways^7^. These large animals also have intact immune systems, and the cellular composition in their TME resembles that of humans^8^. For these reasons, dogs have been regarded as a valuable large animal model for evaluating drug toxicology and efficacy against osteosarcomas in immune-competent conditions.

Protein translation has long been considered as a prime vulnerability in cancers, as several oncogenic drivers are regulated at the level of translation initiation^9^. In nontumorigenic cells, mRNA translation is stringently controlled by the eukaryotic initiation factor 4F (eIF4F) complex, which consists of eIF4A, eIF4E, and eIF4G. As an RNA helicase, eIF4A is critical in unwinding the complex purine-rich 5’-untranslated regions (UTRs) found in the mRNAs that encode growth-promoting kinases, cyclins, transcription factors, and epigenetic regulators^10^. Included among these oncogenic kinases are members of the growth- and survival-promoting PI3K/AKT/mTOR and Ras/RAF/MEK/ERK signaling pathways that are frequently deregulated in cancer, including osteosarcoma^3,4^. These signal transduction cascades converge upon eIF4F and facilitate its assembly and activity, such as promoting the helicase processivity of eIF4A. In cancer cells, this process can establish positive feedback loops whereby oncogenic kinases enhance their own protein synthesis. Thus, blocking eIF4A may simultaneously diminish multiple oncogenic signaling molecules. Indeed, we previously showed that the natural eIF4A inhibitors (−)-rocaglamide [(−)-Roc or RocA] and (−)-didesmethylrocaglamide [(−)-DDR] concurrently reduced the levels of IGF-1R, AKT, ERK1/2, and survivin in osteosarcoma cells and potently suppressed the growth of human osteosarcoma patient-derived xenografts (PDXs)^11^.

Roc and DDR belong to a family of compounds called cyclopenta[*b*]benzofurans (rocaglates or flavaglines). Roc and other bioactive rocaglates selectively bind to eIF4A1/2^12^. Mutational studies in yeast and mouse eIF4A orthologs have identified amino acid substitutions in Phe163 and Ile199 that confer resistance to rocaglates^13,14^. The crystal structure of Roc complexed to eIF4A1 and purine-rich RNA confirmed that these residues are crucial for forming a rocaglate-binding pocket on eIF4A^15^. The high-affinity interactions among Roc, eIF4A, and polypurine RNA sequences effectively prevent intact pre-initiation complexes from successfully translating selected mRNAs. Importantly, the Phe163Leu and Ile199Met substitutions found in the eIF4A homologs of rocaglate-producing *Aglaia* plants prevent Roc toxicity by abolishing Roc-induced polypurine RNA clamping and translation repression. An *Ophiocordyceps* fungus species parasitic on *Aglaia* plants has a glycine substitution at His153 (equivalent to Phe163 in human eIF4A1) that likewise disrupts Roc binding^16^. Collectively, these studies highlight the exquisite specificity of rocaglates as eIF4A inhibitors when they bind to the highly-structured, purine-rich 5’-UTRs of selective mRNAs.

Structure-activity relationship (SAR) analyses from comparing several natural and synthetic rocaglates have identified functional groups important for eIF4A inhibition and positions on the rocaglate scaffold that may be modified to improve drug-like properties^11,17–19^. By side-by-side comparison of 10 naturally-occurring rocaglates, we found (−)-DDR to be the most potent in suppressing the growth of multiple types of sarcoma cells^11^. Here, we showed that human and dog osteosarcoma cells expressed high levels of eIF4A1/2. Genetic depletion of eIF4A1 and/or 2 slowed osteosarcoma cell growth. To advance preclinical development of eIF4A inhibitors, we synthesized racemic (±)-DDR, separated the enantiomers of DDR, and found (−)-DDR to be ≥ 500-fold more active than (+)-DDR in inhibiting the growth of a panel of human and dog osteosarcoma cell lines. We also synthesized two DDR derivatives, (±)-bromo-DDR and (±)-DDR-acetate (Supplementary Figure S1) and found that bromination of DDR at carbon-5 to abolish growth-inhibitory activity, while acetylating DDR at carbon-1 is tolerated. Like DDR and Roc, DDR-acetate promoted G_2_/M arrest and apoptosis in part by decreasing the expression of multiple mitogenic kinases, the STAT3 transcription factor, and the stress-activated protein kinase (SAPK) p38, while enhancing the phosphorylated p38 levels. Since rocaglates can decrease the levels of certain transcription factors^10^, we performed RNA-sequencing (RNA-seq) analysis and identified *RHOB* as a common top upregulated gene, but the Rho inhibitor Rhosin did not enhance the growth-inhibitory activity of (−)-DDR or (−)-Roc. Finally, we showed that these rocaglates potently suppressed the growth of canine osteosarcoma PDXs.

## Results

### Human bone sarcoma cells express high levels of eIF4F components, particularly eIF4A2.

**Figure 1:**
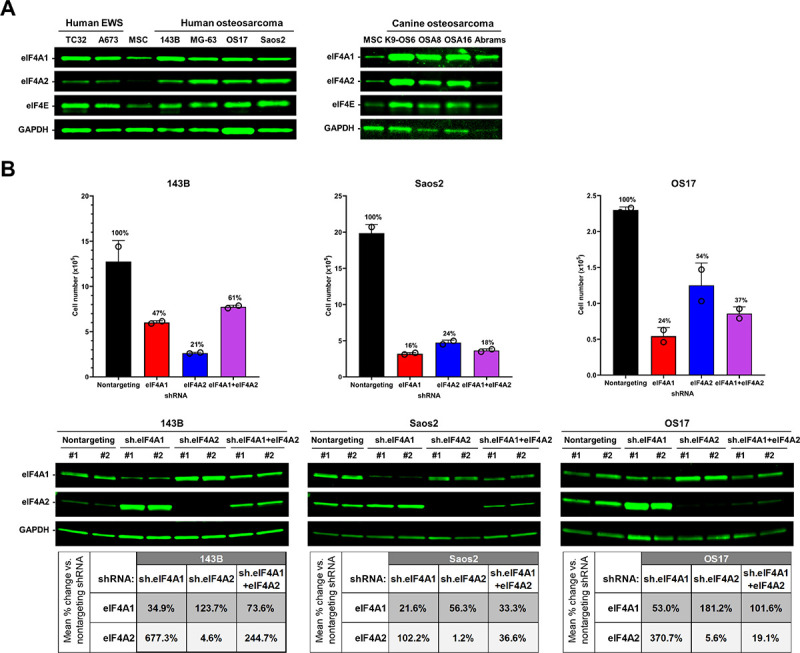
Osteosarcoma and Ewing sarcoma cells express high levels of eIF4F components, which are important for tumor cell proliferation. (**A**) Western blots of lysates from human osteosarcoma and Ewing sarcoma (EWS) cell lines and canine osteosarcoma cell lines were probed for eIF4A1, eIF4A2, eIF4E, and GAPDH as a loading control. Human MSCs were used for comparison. (**B**) Genetic knockdown of eIF4A impairs osteosarcoma cell proliferation. Human osteosarcoma 143B, Saos2, and OS17 cells were infected with 10 MOI of lentiviral particles expressing a nontargeting shRNA (negative control) or shRNAs targeting eIF4A1 and/or eIF4A2. When the nontargeting-shRNA transduced wells reached confluence, cells were trypsinized and counted on a hemocytometer. The bar graphs depict the total number of cells, expressed as the mean + SD of duplicate wells. The relative numbers of targeted shRNA-transduced cells, expressed as a percentage of nontargeting shRNA-transduced cells (defined as 100%), are listed above the bars (upper panels). After counting, the harvested cells were lysed and analyzed by Western blots to detect eIF4A1 and eIF4A2 expression with GAPDH as a loading control. The relative band intensity of eIF4A1 or eIF4A2 was calculated by normalizing to that of GAPDH in each sample. These normalized values from biological replicates were averaged, and the mean changes in protein expression relative to the cells transduced with a nontargeting control shRNA estimated. The tables display the mean percent change in normalized levels of eIF4A1 and eIF4A2 protein after gene silencing (bottom panels).

We previously demonstrated that malignant peripheral nerve sheath tumor (MPNST), another aggressive type of sarcoma, overexpress components of the eIF4F complex^20^. To determine if this feature is characteristic of other types of sarcomas, we assessed the protein levels of both eIF4A isoforms 1 and 2 and eIF4E in four human and four canine osteosarcoma and two human Ewing sarcoma cell lines. Compared to MSCs, which have been postulated to be the cell-of-origin for bone sarcomas^21^, all human osteosarcoma and Ewing sarcoma cell lines examined overexpressed both eIF4A1 and 2 and eIF4E (Fig. 1A). In particular, the amounts of eIF4A2 were substantially higher in these sarcoma cells relative to MSCs. Similarly, all four dog osteosarcoma cell lines studied expressed high levels of eIF4A1/2 and eIF4E. These results indicate that osteosarcoma and Ewing sarcoma cells frequently overexpress eIF4F components.

### Silencing of EIF4A1 or EIF4A2 reduces osteosarcoma cell growth.

To examine whether eIF4A1/2 are important for osteosarcoma cell proliferation, we performed shRNA-mediated silencing in three human osteosarcoma cell lines. *EIF4A1* knockdown in Saos2, 143B, and OS17 cells reduced the eIF4A1 protein levels by 78, 65, and 47%, respectively (Fig. 1B). As was previously reported by us and others^20,22^, depletion of eIF4A1 in osteosarcoma cells caused a feedback-mediated compensatory increase in eIF4A2 protein levels, particularly in 143B and OS17 cells. *EIF4A2* knockdown in the same three osteosarcoma cell lines caused a 94–99% reduction of eIF4A2 protein levels. Also, depletion of eIF4A2 was associated with increased eIF4A1 protein levels in OS17 and 143B cells. However, simultaneous knockdown of both *EIF4A1* and *EIF4A2* resulted in higher expression levels of eIF4A1/2 proteins in all three osteosarcoma cell lines, compared to *EIF4A1* or *EIF4A2* knockdown alone. It is possible that this effect may be due to feedback compensation and warrants further investigation.

Importantly, silencing *EIF4A1* or *EIF4A2* slowed the growth of all three osteosarcoma cell lines, and the amount of growth inhibition correlated with the magnitude in the decrease of target protein levels (Fig. 1B). Co-silencing of both *EIF4A1* and *EIF4A2* also resulted in reduced cell growth in all three osteosarcoma cells, but the effect was slightly less than that by *EIF4A1* silencing alone. Additionally, the effect of growth inhibition by combined *EIF4A1* and *EIF4A2* knockdown was less than that by *EIF4A2* knockdown in 143B cells. These attenuated antiproliferative effects may be due to less reduction in the levels of eIF4A1 and eIF4A2 proteins in double-knockdown cells. Nonetheless, the results indicate that both eIF4A1 and eIF4A2 are important for osteosarcoma cell proliferation.

### (−)-DDR, (−)-Roc, and the DDR derivative DDR-acetate potently inhibit proliferation of human and dog osteosarcoma cells.

**Figure 2:**
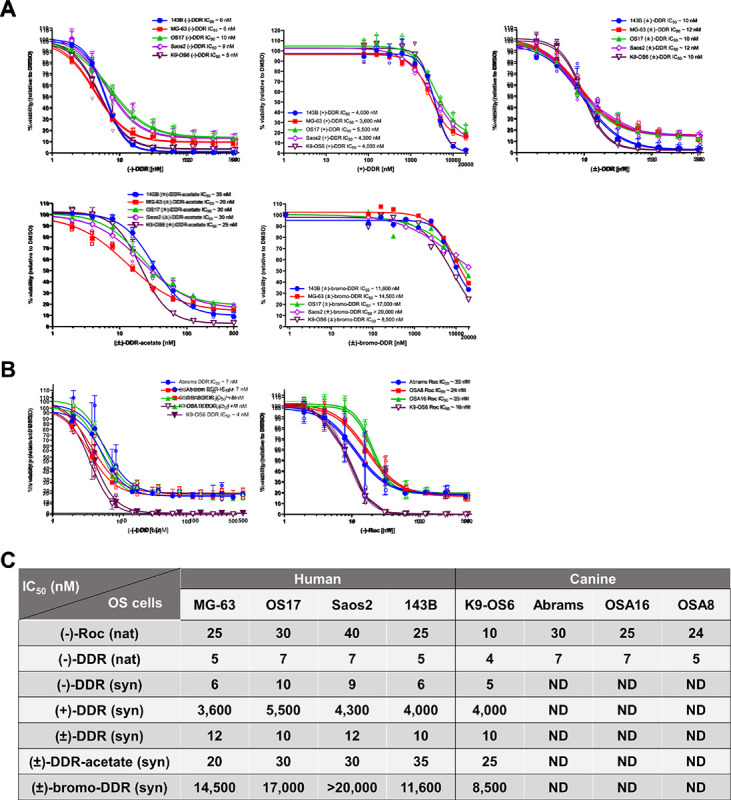
(−)-Roc, (−)-DDR and racemic (±)-DDR and (±)-DDR-acetate potently suppress proliferation of human and dog osteosarcoma cells, while (+)-DDR and (±)-bromo-DDR have greatly attenuated activity. (**A**) Actively-growing human osteosarcoma 143B, MG-63, OS17, and Saos2 and canine osteosarcoma K9-OS6 cells were treated with (−)-DDR, (+)-DDR, (±)-DDR, (±)-DDR-acetate, or (±)-bromo-DDR as described in Methods. Cell proliferation was estimated as a percentage of the DMSO vehicle control designated as 100%. Graphs depict the means and SDs from 2 or 3 independent experiments. (**B**) Dog osteosarcoma Abrams, OSA8, OSA16, and K9-OS6 cells were treated with enantiopure (−)-Roc or (−)-DDR purified from *Aglaia* extracts, and cell proliferation was estimated as above. (**C**) Summary of the mean IC_50_ values for the indicated natural (nat) and synthetic (syn) rocaglates in various human and dog osteosarcoma (OS) cell lines. ND, not determined.

Studies have shown that bioactive rocaglates exert their anti-proliferative effects through eIF4A inhibition^14,15^. We also found that (−)-DDR and (−)-Roc potently suppress the growth of multiple types of sarcoma cells with (−)-DDR consistently showing 2 ~ 3-fold higher activity^11^. SAR analysis revealed that the C-8b hydroxy group on the cyclopenta[*b*]benzofuran core is essential for growth-inhibitory activity while modifying the C-2 and C-6 positions may enhance this activity. To expand SAR analysis, we synthesized racemic (±)-DDR and chirally separated racemic (±)-DDR into (−)-DDR and (+)-DDR. As expected^11^, enantiopure (−)-DDR and (−)-Roc inhibited the growth of all four tested human osteosarcoma cell lines with IC_50_ values of 5~7 nM and 25 ~ 40 nM, respectively (Fig. 2). The potent anti-proliferative activities of (−)-DDR and (−)-Roc were also observed in four dog osteosarcoma cell lines with IC_50_ values ranging from 4~7 nM for (−)-DDR and 10~30 nM for (−)-Roc (Fig. 2B). Intriguingly, when compared to (−)-DDR, racemic (±)-DDR was generally half as potent at reducing osteosarcoma cell proliferation (Fig. 2A). In contrast, the growth-inhibitory activity of (+)-DDR in osteosarcoma cells was substantially reduced with IC_50_ values of 3.6 ~ 5.8 μM. These findings support the importance of the (−) configuration of DDR in growth suppression.

To examine the effects of modifying the C1 and C5 positions, we synthesized DDR-acetate, which is acetylated at C1, and bromo-DDR, which is brominated at C5 of the A-ring (Supplementary Figure S1). In various human and dog osteosarcoma cell lines tested, (±)-DDR-acetate had an IC_50_ value (20 ~ 35 nM) roughly 2–3-fold higher than that of (±)-DDR (10–12 nM) (Figs. 2A,C). In contrast, (±)-bromo-DDR exhibited little growth-inhibitory activity, with an IC_50_ value ranging from 8.5 μM in the dog PDX-derived K9-OS6 cell line to > 20 μM in human Saos2 cells. These results indicate that acetylating the hydroxyl group at C1 of DDR is well-tolerated, while modifying the C5 position on A-ring aryl group substantially reduces antiproliferative activity.

### (±)-DDR-acetate, like (−)-DDR and (−)-Roc, induces G_2_/M arrest and apoptosis in human and canine osteosarcoma cells.

**Figure 3:**
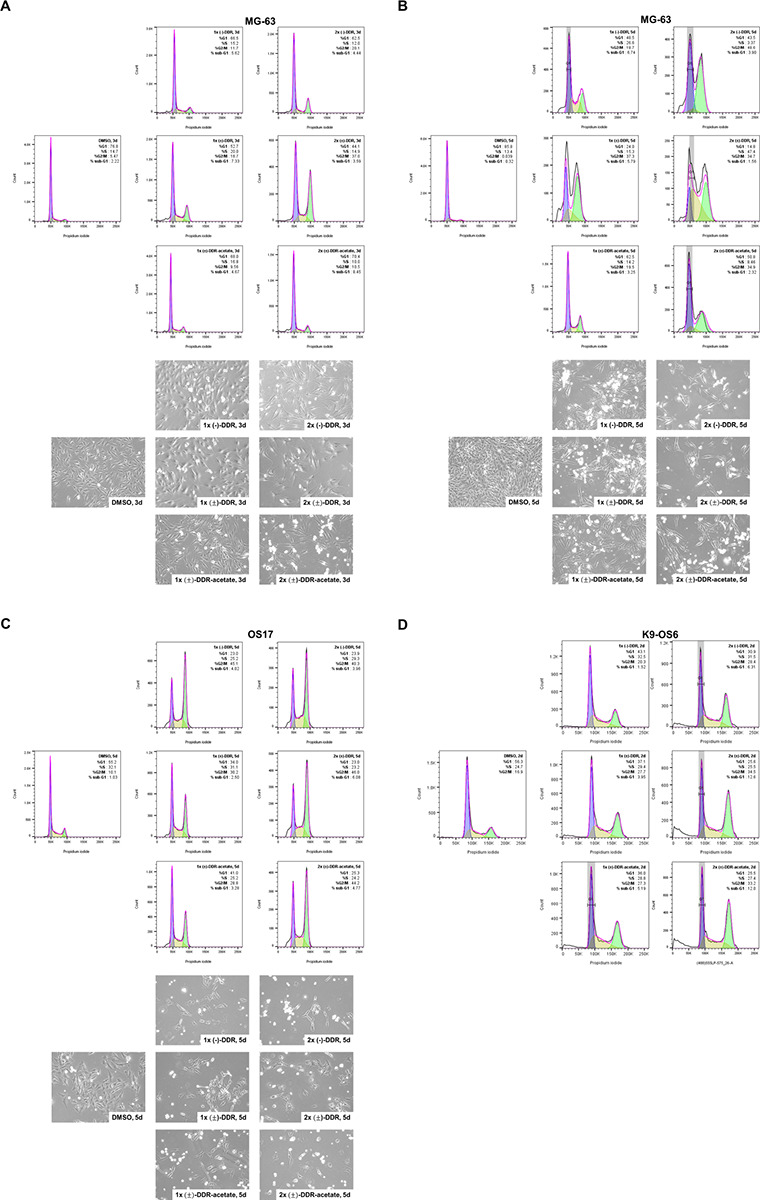
(−)-DDR, (±) DDR, and DDR-acetate induce G_2_/M arrest and cell death in human and dog osteosarcoma cells. MG-63 (**A** and **B**) and OS17 (**C**) human osteosarcoma cells were treated with 1x and 2x IC_50_ of (−)-DDR, (±)-DDR, or (±)-DDR-acetate for three (**A**) or five days (**B** and **C**). Treated cells were fixed, stained with propidium iodide, and analyzed for cell cycle distribution by flow cytometry. Shown are the cell cycle histograms with the % of cells in G_1_, S, and G_2_/M phases, as well as the sub-G_1_ fraction. Below the histograms are representative phase contrast micrographs of cells from each treatment prior to flow analysis. (**D**) K9-OS6 dog osteosarcoma cells were treated for 2 days with 1x and 2x IC_50_ of (−)-DDR, (±)-DDR, or (±)-DDR-acetate, followed by flow analysis. Cell cycle histograms were generated as described above.

To examine the mechanisms of growth inhibition, we first compared the effects of (±)-DDR-acetate, (±)-DDR, and (−)-DDR on cell cycle distribution of human and dog osteosarcoma cells. An increase in the G_2_/M population was observed in MG-63 cells treated with 1x and 2x IC_50_ concentrations of all three rocaglates for three or five days (Figs. 3A,B). Increased sub-G_1_ fractions were also observed in all three rocaglate-treated MG-63 cells, suggesting induction of cell death. Similarly, we detected prominent G_2_/M arrest and an increase in the sub-G_1_ fraction in OS17 cells treated with all three rocaglates (Fig. 3C). Like in human osteosarcoma cells, all three rocaglates also increased the G_2_/M and sub-G_1_ populations in K9-OS6 cells treated for two days (Fig. 3D).

**Figure 4:**
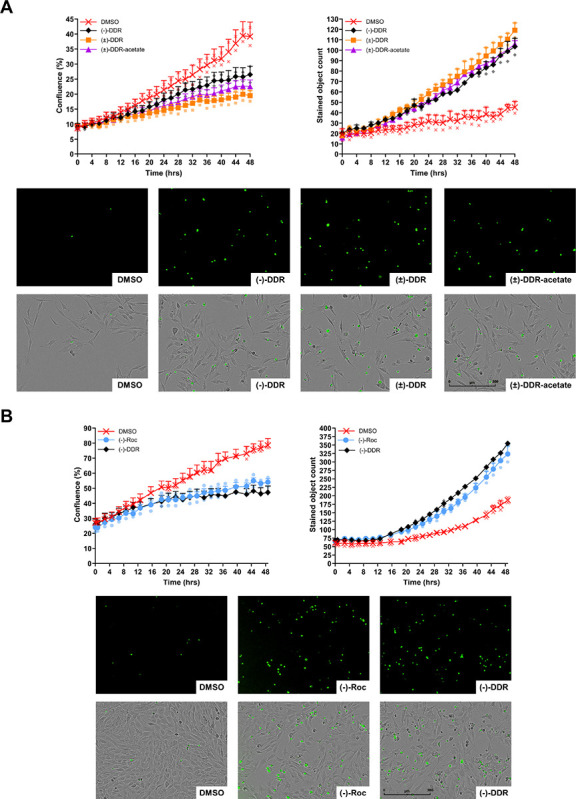
(±)-DDR-acetate, (±)-DDR, (−)-DDR, and (−)-Roc inhibit proliferation of osteosarcoma cells by rapidly inducing caspase-3/7 cleavage, indicative of apoptosis. For dynamic quantitation of cell confluency and caspase-3/7 cleavage, K9-OS6 dog osteosarcoma cells were treated in duplicate with 2x IC_50_ of (−)-DDR, (±)-DDR, or (±)-DDR-acetate and imaged in real-time on the Incucyte^®^ live-cell imaging system as described in Methods. Graphs depict the 48-hour time course showing the percent confluency (left) and caspase-3/7 labeling (right) of treated cells (means+SEMs) (**A**). Below the graphs are representative green fluorescent (indicating caspase-3/7 activation) and merged fluorescent and phase contrast micrographs of treated cells. Similarly, K9-OS6 cells were treated in duplicate with 2x IC_50_ of (−)-Roc or (−)-DDR and evaluated over 2 days for confluency and caspase-3/7 activation using the Incucyte live-cell imaging (**B**).

To determine whether induction of cell death by DDR-acetate, (±)-DDR, and (−)-DDR is due to apoptosis, we performed Incucyte^®^ live cell imaging combining with caspase-3/7 cleavage assay in K9-OS6 cells treated with each rocaglate. Growth inhibition was observed with concomitantly increased caspase-3/7 cleavage in cells treated with all three rocaglates, when compared to DMSO-treated controls (Fig. 4A). Significant increases in caspase cleavage were seen, starting from ~12 hours of treatment and becoming more pronounced thereafter. Like (−)-DDR, (−)-Roc also inhibited the growth of K9-OS6 cells while simultaneously enhancing caspase-3/7 cleavage (Fig. 4B). These results were corroborated by flow cytometry analysis showing prominent increases in the G_2_/M and sub-G_1_ populations in DDR or Roc-treated cells (Supplementary Figure S2).

Together, these results indicate that apoptosis induction contributes to the antiproliferative effects of DDR-acetate, (±)-DDR, (−)-DDR, and (−)-Roc in human and dog osteosarcoma cells.

### (±)-DDR-acetate also suppress multiple signaling molecules important for osteosarcoma growth while activating cellular stress and DNA damage response pathways.

**Figure 5:**
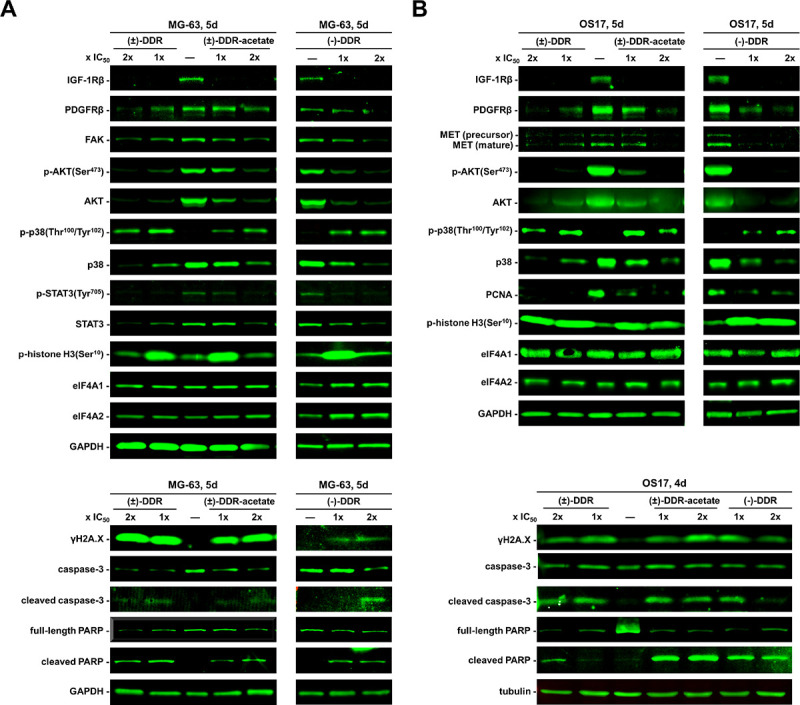
(−)-DDR, (±)-DDR, and DDR-acetate diminish the expression of multiple mitogenic kinases and concomitantly enhanced the levels of markers for cell stress, apoptosis, and DNA damage response in osteosarcoma cells. MG-63 (**A**) and OS17 (**B**) were treated 4 or 5 days (d) with 1x or 2x IC_50_ of (−)-DDR, (±)-DDR, or (±)-DDR-acetate. Equal amounts of protein from whole cell lysates were used in immunoblotting for the indicated proteins. GAPDH and tubulin were used as loading controls.

We previously showed that (−)-Roc and (−)-DDR inhibit the expression of several mitogenic kinases in sarcoma cells^11^. To examine whether the growth-inhibitory activity of (±)-DDR-acetate acted through similar molecular mechanisms, we performed Western blot analysis on human osteosarcoma cells treated with (±)-DDR-acetate, (±)-DDR, or (−)-DDR. All three rocaglates markedly diminished the levels of IGF-1R and AKT (total and phospho-AKT [p-AKT]) in both MG-63 and OS17 cells (Fig. 5). Also, reduced expression of PDGFRβ, p38 SAPK, FAK, and the transcription factor STAT3 (total and p-STAT3) was observed in treated MG-63 cells. In addition, these rocaglates decreased the MET levels in OS17 cells. Intriguingly, while p38 expression was diminished, phosphorylated p38 was notably enhanced, and the γH2A.X levels were elevated in treated MG-63 and OS17 cells, indicating activation of genotoxic stress signaling and DNA damage responses. In line with induction of G_2_/M arrest, we detected abundant p-histone H3, a G_2_/M marker, particularly in cells treated with 1x IC_50_. Further, all three rocaglates increased cleavage of caspase-3 and PARP in both osteosarcoma cell lines, signifying enhanced apoptosis. Notably, the protein levels of housekeeping proteins GAPDH and tubulin, as well as eIF4A1, and eIF4A2 were unaffected by these rocaglate treatments. These results reinforce that eIF4A inhibition does not indiscriminately inhibit translation initiation but selectively affects transcripts encoding mitogenic proteins.

**Figure 6:**
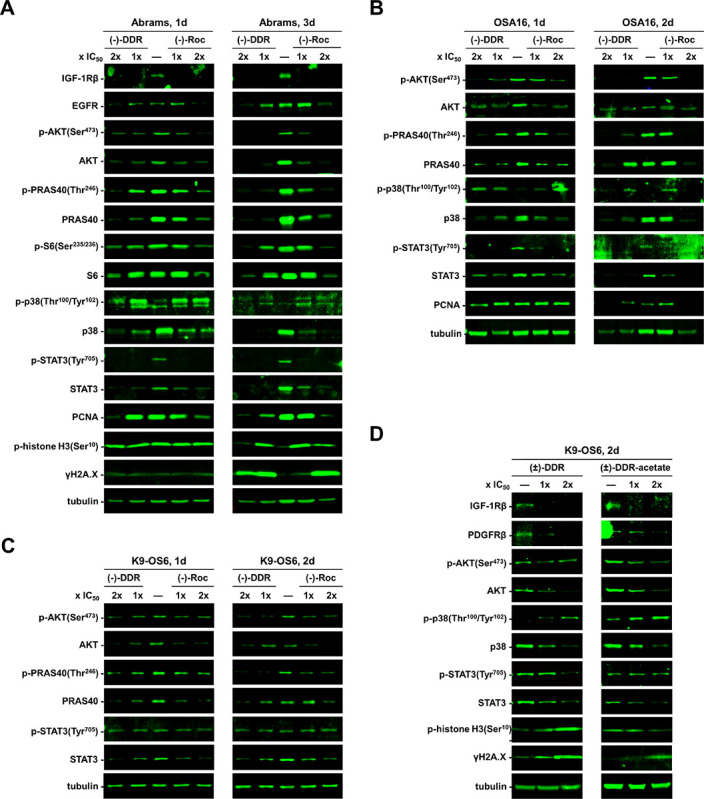
Canine osteosarcoma cells treated with (−)-DDR, (±)-DDR, DDR-acetate, or (−)-Roc also exhibit reduced expression of mitogenic kinases but enhanced levels of markers for cell stress, apoptosis, and DNA damage response. Abrams (**A**), OSA16 (**B**), and K9-OS6 (**C**) dog osteosarcoma cells were treated with 1x and 2x IC_50_ of (−)-DDR or (−)-Roc for the indicated days (d). Equal amounts of protein lysates were immunoblotted and probed for the indicated signaling proteins with tubulin as the loading control. Also, immunoblots of protein lysates from K9-OS6 cells treated with 1x and 2x IC_50_ of (±)-DDR or (±)-DDR-acetate were performed and probed for various signaling molecules (**D**).

Similarly, we treated three dog osteosarcoma cell lines with (−)-Roc and (−)-DDR. Both rocaglates diminished the expression of IGF-1R, EGFR, total AKT, p-AKT, and its downstream targets PRAS40 and S6 in Abrams cells (Fig. 6A). Additionally, these rocaglates reduced the levels of total STAT3, p-STAT3, and PCNA. As in Abrams cells, (−)-Roc and (−)-DDR decreased the expression of AKT, p-AKT, its downstream substrate PRAS40, as well as STAT3 and p-STAT3 in OSA16 and K9-OS6 cells (Figs. 6B,C). Like in human osteosarcoma cells, (−)-Roc and (−)-DDR enhanced the phosphorylation of p38 in Abrams and OSA16 cells, despite decreases in total p38 levels (Figs. 6A,B). Also, we detected increased p-histone H3 and γH2A.X expression in treated Abrams cells (Fig. 6A). Further, both (±)-DDR-acetate and (±)-DDR reduced the expression of IGF-1R, PDGFRβ, AKT, and STAT3, while elevating phospho-p38 and γH2A.X levels in K9-OS6 cells (Fig. 6D).

Overall, these data indicate that (±)-DDR-acetate, (±)-DDR, (−)-DDR, and (−)-Roc suppress human and dog osteosarcoma cell growth by decreasing the levels of key growth-promoting molecules and inducing cell stress and DNA damage responses, ultimately leading to apoptosis.

### (−)-DDR and (−)-Roc potently suppress tumor growth in a canine osteosarcoma PDX model.

**Figure 7:**
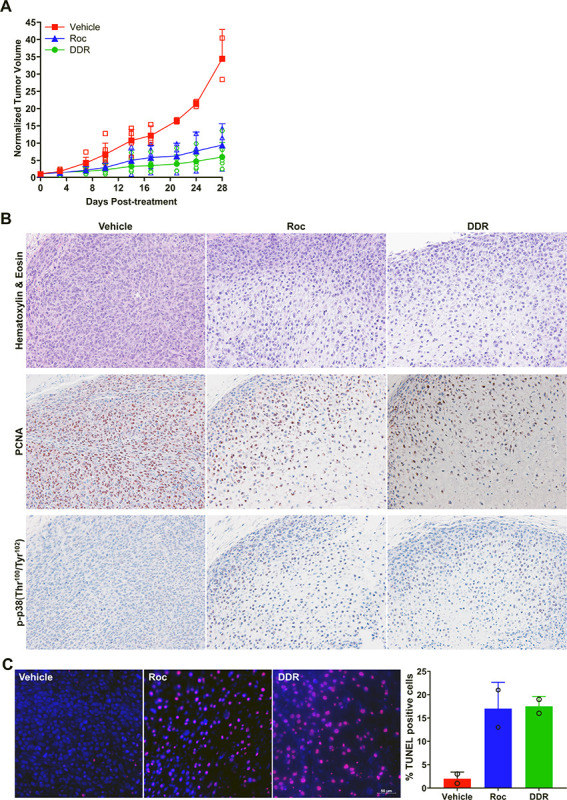
(−)-DDR and (−)-Roc effectively suppress tumor growth and promote apoptosis in a canine osteosarcoma PDX model. (**A**) NSG mice bearing actively-growing OSU-K9-OS6 PDXs were treated with DDR, Roc, or vehicle, and tumor volumes measured according to Methods. The mean fold-increase in normalized tumor volume over time was calculated by dividing each individual tumor volume to its value at the start of treatment set as 1. Shown are means+SDs. (**B**) Representative images of vehicle-, Roc-, and DDR-treated tumor sections stained with hematoxylin & eosin (top panels), or with the antibody against PCNA (middle panels) or p-p38 (bottom panels). (**C**) Tumor sections were also subjected to TUNEL staining and showed that DDR- and Roc-treated tumors had increased numbers of TUNEL-positive nuclei (red). DAPI stained nuclei in blue. The percentage of TUNEL-positive cells was estimated by counting ≥300 nuclei across 2 high-power fields in each treated tumor using the Cell Counter plugin of Fiji (https://fiji.sc). Bar graph shows the means and SDs of % TUNEL-labeled nuclei for each treated tumor.

Since dogs frequently develop osteosarcomas with molecular signatures similar to human tumors^6,7^, we evaluated the *in vivo* efficacy of (−)-DDR and (−)-Roc using the OSU-K9-OS6 dog osteosarcoma PDX model. Compared to vehicle controls, (−)-DDR and (−)-Roc significantly suppressed tumor growth by ~ 81% and 73%, respectively, over four-week treatment (Fig. 7A). While vehicle-treated OSU-K9-OS6 PDXs exhibited sheet-like growth with high cellularity and active mitotic figures, (−)-DDR and (−)-Roc-treated tumors showed degenerative changes with substantially reduced cellularity, and immunohistochemistry (IHC) analysis confirmed decreased labeling of the proliferation marker PCNA and increased staining of phospho-p38 (Fig. 7B). Consistent with increased cleaved caspase-3/7 activities *in vitro* (Fig. 4B), TUNEL and cleaved caspase-3 staining demonstrated that both rocaglates greatly enhanced apoptosis in treated tumors (Figs. 7C and Supplementary Figure S3). These results indicate that (−)-DDR and (−)-Roc also exhibit potent anti-tumor effects in dog osteosarcomas.

### (−)-DDR and (−)-Roc treatment upregulate genes and pathways linked to inflammatory responses, cell stress and death, and growth-related signaling.

**Figure 8:**
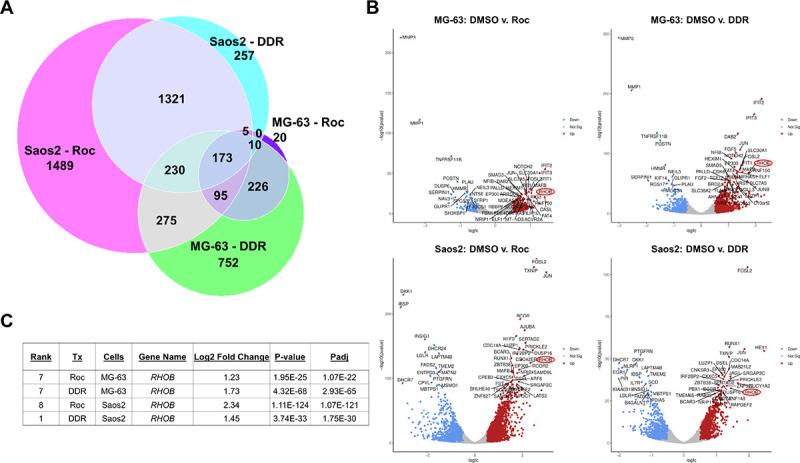
RNA-seq analysis of (−)-DDR- or (−)-Roc-treated osteosarcoma cells identifies top common differentially expressed genes and pathways. MG-63 and Saos2 cells were treated 24h with 1x IC_50_ of (−)-DDR or (−)-Roc and analyzed by RNA sequencing. (**A**) Venn diagram of significant protein-coding DEGs (Padj ≤ 0.05; absolute log2-FC=0.5) in treated osteosarcoma cells, showing that 173 DEGs are shared among all rocaglate-treated cells. (**B**) Volcano plots depicting the top-50 protein-coding DEGs identified in (−)-DDR- or (−)-Roc-treated MG-63 and Saos2 cells. Among these top DEGs, *RHOB* (circled in red) was detected in both osteosarcoma cell lines treated with either rocaglate. Blue = down-regulated; red = up-regulated; grey = non-significant. (**C**) Table summarizing the ranking of *RHOB* among the top-50 DEGs, the log2 fold-change, and the significance values in (−)-DDR- or (−)-Roc-treated MG-63 or Saos2 cells.

Prior studies demonstrate that the transcripts encoding transcription factors like MYC and JUN have long purine-rich 5’-UTRs and are sensitive to inhibition by Roc and related rocaglates^23,24^. As we found that DDR and Roc also decreased the STAT3 levels in osteosarcoma cells (Fig. 5), we investigated the effects of these rocaglates on the transcriptomes of MG-63 and Saos2 cells treated with (−)-DDR or (−)-Roc for one day by RNA-seq. Comparing the set of protein-coding DEGs in rocaglate vs. DMSO-treated cells, we identified 173 differentially-expressed transcripts in both rocaglate-treated MG-63 and Saos2 cells (Fig. 8A and Supplementary Table S1). Of these shared transcripts, 96% were similarly up- or downregulated by (−)-DDR and (−)-Roc treatment of MG-63 and Saos2 cells, including 121 upregulated (~ 70%) and 45 downregulated (~ 26%) DEGs (Supplementary Figure S4). Of these 173 shared DEGs, many were also found among the top-50 protein-coding DEGs in both osteosarcoma cell lines treated with either (−)-DDR or (−)-Roc (compare Fig. 8B with Supplementary Table S1). These include upregulation of transcripts encoding the RhoB small GTPase, the dual-phosphatase DUSP16 which dephosphorylates and inactivates ERK and JNK, and the transcription factors JUN, FOSL2, MAFB, and HEY1; several of these proteins are important for osteosarcoma cell growth and differentiation.

By GSEA, we identified 21 up- and 11 down-regulated pathways shared in (−)-DDR or (−)-Roc treated MG-63 and Saos2 cells (Supplementary Figures S5 and S6 and Supplementary Table S2). The common top upregulated pathways included those involved in inflammatory responses and cytokine signaling (e.g., interferon-α and -γ responses and TNFα signaling), stress-related responses (e.g., UV response and hypoxia), G_2_/M checkpoint and apoptosis (e.g., p53 pathway, and mitotic spindle), and growth- associated NOTCH, WNT/β-catenin, and TGFβ signaling. The common top downregulated pathways were related to lipid metabolism (e.g., cholesterol homeostasis and fatty acid metabolism), E2F targets, mTORC1 signaling, glycolysis, and oxidative phosphorylation. These results are in line with decreased growth-promoting signaling and increased cell stress, DNA damage response, and apoptosis in (−)-DDR- or (−)-Roc-treated cells (Figs. 5 and 6).

Although (−)-DDR or (−)-Roc potently suppressed osteosarcoma growth, they did not eliminate all tumor cells (Fig. 7). Since *RHOB* was identified among the top ten upregulated DEGs in both MG-63 and Saos2 treated with either rocaglate (Figs. 8B,C), we tested whether increased *RHOB* expression provided any survival benefits. All four human osteosarcoma cell lines were tested with (−)-DDR and (−)-Roc in a dose-matrix combination with the Rho inhibitor Rhosin^25^. However, Rhosin alone had little or no growth-inhibitory activity even at 50 μM (Supplementary Figure S7), and combining Rhosin with (−)-DDR or (−)-Roc did not give rise to enhanced growth inhibition according to Bliss synergy scores. These results suggest that *RHOB* upregulation in (−)-DDR or (−)-Roc treated osteosarcoma cells does not confer a survival benefit.

## Discussion

With the standard-of-care that has not changed over the last four decades, patients with osteosarcoma have not yet benefited from recent advances in genomic profiling and therapeutic development. Although patients with localized disease have a 70% five-year survival rate, this comes at the cost of severely decreased quality-of-life. Radical limb amputation is frequently used, and the harsh multi-cytotoxic chemotherapy incurs acute and chronic systemic toxicities^26,27^. Further, patients suffering relapse and those with metastatic disease have an especially bleak prognosis. Efforts to identify effective drugs have been impeded by both intra- and inter-tumor heterogeneity of this aggressive bone cancer^1,3,4^. A therapy that simultaneously inhibits multiple oncogenic drivers is likely to be needed to eradicate osteosarcoma.

To sustain uncontrolled growth, cancer cells often exhibit enhanced protein translation by upregulation of the translation machinery. Previously, we found that MPNST, another type of sarcoma, frequently over-expresses the three eIF4F components^20^. Along with this line, we showed that both human and dog osteosarcoma cells, as well as Ewing sarcoma cells expressed high levels of eIF4A1, eIF4A2, and eIF4E (Fig. 1). Also, silencing *EIF4A1* or *EIF4A2*impeded osteosarcoma cell proliferation. However, the role of eIF4A2 in cell proliferation may depend on the cell type. A previous study showed that while *EIF4A1* knockdown reduces the growth of HeLa cells, depletion of eIF4A2 has no effects^28^. On the contrary, silencing *EIF4A2* in embryonic stem cells compromised their proliferation and disrupted the stem cell maintenance program^29^. Also, *Eif4a2* knockout is embryonically lethal in mice. Our finding that silencing *EIF4A1* and/or *EIF4A2* impeded the growth of multiple osteosarcoma cell lines indicate that both eIF4A isoforms are required for osteosarcoma cell proliferation. Curiously, genetic suppression of *EIF4A1* resulted in increased eIF4A2 levels in 143B and OS17 cells, likely due to increased transcription of *EIF4A2*as previously reported^22^. Likewise, we observed modestly increased eIF4A1 expression in osteosarcoma cells after *EIF4A2*silencing, suggesting potential feedback interactions between *EIF4A1* and *EIF4A2*. Together, out results further emphasize the importance of eIF4A in sarcoma growth.

Previous studies have established the specificity of Roc and related rocaglates as eIF4A inhibitors using biological and biochemical assays and X-ray crystallography^12,14,15^. To further understand the SAR of rocaglates, we confirmed the importance of chirality, as the (+)-DDR enantiomer is 500-to-800-fold less bioactive than (−)-DDR in various osteosarcoma cell lines (Fig. 2). Consistently, racemic (±)-DDR was generally half as potent at reducing osteosarcoma cell proliferation as (−)-DDR. We also observed the importance of the C5 position of the A-ring in rocaglate as bromination of this position, as in (±)-bromo-DDR, almost abolished its antiproliferative activity. Based on the crystal structure of (−)-Roc bound to eIF4A1 and polypurine RNA^15^, we posit that the bulky C5-bromine prevents the A-ring from stacking in parallel with the polypurine tract. Interestingly, functionalizing the C1 position with an acetyl group, as in (±)-DDR-acetate, was well-tolerated, with only mildly decreased growth-inhibitory activity compared to (±)-DDR. An identical substitution in a natural rocaglamide analog, (−)-Roc-AB (1-*O*-acetylrocaglamide), was reported to show a similar IC_50_ to (−)-Roc and inhibit leukemia cell growth^30^. However, aza-rocaglamide with a C1-substituted aminomethyl group oriented in *syn* to the 8b-hydroxy group exhibited 30-fold greater antiproliferative activity than the stereoisomer with the *anti-*configuration^31,32^. Curiously, our DDR-acetate has the same 1,8b-*anti*-configured acetyl group as that in (−)-Roc-AB. Thus, it will be worthwhile to see if functionalizing the C1 position with an acetyl or aminomethyl group with the 1,8b-*syn*-configuration improves the antiproliferative effects of DDR.

Mechanistically, (±)-DDR-acetate, like (−)-DDR and (−)-Roc, diminished the expression of several receptor tyrosine kinases (RTKs) important for osteosarcoma growth, including IGF-1R, PDGFRβ, EGFR, and MET, in human and dog osteosarcoma cells (Figs. 5 and 6). Additionally, the levels of other mitogenic kinase, such as AKT and FAK, and the transcription factor STAT3, which is important for osteosarcoma cell growth, metabolism, survival, and metastatic behavior^33^, were greatly reduced. Moreover, these rocaglates induced G_2_/M arrest as evidenced by increased G_2_/M population and phospho-histone H3 expression in treated osteosarcoma cells. Also, they elevated the levels of γH2A.X and cleaved caspase 3 and PARP (Figs. 3–6), suggestive of induction of DNA damage responses and apoptosis.

Interestingly, treatment of human and dog osteosarcoma cells with (−)-Roc-, (−)-DDR-, (±)-DDR, and (±)-DDR-acetate increased phosphorylated and activated p38 SAPK (Figs. 5 and 6), despite marked declines in the total p38 protein level, consistent with translation inhibition. Presently, the mechanism by which rocaglate treatments activate p38 is not understood. As a mediator of intrinsic apoptosis, p38 is activated by a variety of stimuli, including oxidative and genotoxic stressors^34^. Activated p38 can phosphorylate transcription factors, such as p53, and initiate caspase activation to execute apoptosis. Our GSEA of RNA-seq data substantiates these findings, with several significantly upregulated pathways including those associated with UV responses, hypoxia, p53 pathway, mitotic spindle assembly, G_2_/M checkpoint, and apoptosis in both (−)-DDR and (−)-Roc treated MG-63 and Saos2 cells (Supplementary Figure S5 and Supplementary Table S2). Thus, it is possible that rocaglate treatments induce these stressors, leading to p38 activation. Curiously, we also identified several pathways that were associated with inflammatory responses and cytokine signaling, especially activation of type I interferon-stimulated genes (ISGs), among the common top upregulated pathways. Induction of ISGs is linked to cell stress responses, but they may also have effects on the tumor immune microenvironment when osteosarcomas are grown in immune-competent conditions^35^.

Importantly, we showed that (−)-DDR and (−)-Roc potently suppress tumor growth in a canine osteosarcoma PDX model (Fig. 7). Treated tumors exhibited greatly reduced cellularity with degenerative changes and, consistent with *in vitro* findings, had increased phospho-p38 expression and high apoptotic labelling. Together with the anti-tumor activity of (−)-Roc in human sarcoma PDX models^11^, these results indicate that these rocaglates, as eIF4A inhibitors, can be used to treat both human and dog osteosarcomas. It should be noted that a synthetic derivative of Roc, eFT-226 (Zotatifin)^36,37^ has demonstrated safety and tolerability and is now in phase 2 clinical trial to treat patients with K-Ras or RTK-driven advanced breast cancer and non-small cell lung carcinoma (ClinicalTrials.gov identifier NCT04092673). Thus, a clinical trial in canine patients with spontaneous osteosarcomas and other soft-tissue sarcomas is warranted.

However, (−)-DDR or (−)-Roc did not eliminate all osteosarcoma cells (Fig. 7), suggesting possible emergence of compensatory survival mechanisms that would require a combination therapy. Our DEG analysis identified *RHOB,* which encodes a small GTPase associated with cell motility, membrane trafficking, and cell proliferation^38^, within the top 10 upregulated genes in both (−)-DDR- and (−)-Roc-treated MG-63 and Saos2 cells (Fig. 8C). Despite this finding, we did not observe enhanced growth suppression in osteosarcoma cells after combined inhibition of eIF4A and Rho (Supplementary Figure S7). Previously by CRISPR screening, a novel interaction between rocaglates and the KEAP1-CUL3-NRF2 axis was identified^39,40^. Under normal conditions, KEAP1, which binds to CUL3 and NRF2, promotes NRF2 ubiquitination and proteasomal degradation^41^. Upon exposure to reactive oxygen species or other stressors, KEAP1 undergoes conformational changes, liberating NRF2 from the complex. Free NRF2 translocates to the nucleus where it heterodimerizes with MAF proteins and binds to antioxidant responsive elements to activate transcription of antioxidant and metabolic genes. Some of these NRF2-activated gene products can enhance translation of eIF4A-dependent transcripts. Intriguingly, we detected *MAFB* as one of the common top upregulated protein-coding genes, while the RNA levels of *NFE2L2,* which encodes NRF2, were only slightly elevated in (−)-DDR- and (−)-Roc-treated osteosarcoma cells (Fig. 8 and Supplementary Tables S1 and S3). It will be interesting to see if MAFB provides any survival benefits to osteosarcoma cells.

Furthermore, we identified the NOTCH, Hedgehog, TGFβ, and β-catenin pathways among the common top upregulated pathways in (−)-DDR- and (−)-Roc-treated MG-63 and Saos2 cells. These signaling pathways have been reported to drive osteosarcoma cell proliferation, and high expression of proteins in these pathways correlates with tumor aggressiveness and patient survival^42^. Also, signaling from TGFβ receptor family members can enhance osteosarcoma malignant behavior, with tumors expressing high *TGFB1* levels being more likely to respond poorly to chemotherapy^43^. Among the TGFβ signaling gene set, the signal transducers for TGFβ receptors *SMAD1, SMAD3,* and *SMAD6* and the bone-morphogenetic protein receptor 2 *(BMPR2)* were highly upregulated in rocaglate-treated osteosarcoma cells (Supplementary Table S2). Studies have shown that the SMAD-dependent TGFβ and BMP signaling is important for bone differentiation and formation. There are cross-talks between TGFβ/BMP and WNT signaling and between WNT and NOTCH signaling^44^. Thus, it is tempting to speculate that upregulation of these pathways may provide compensatory survival benefits in rocaglate-treated osteosarcoma cells. We are presently testing whether combining the inhibitors of these pathways synergizes with (−)-DDR and (−)-Roc to kill osteosarcoma cells.

In summary, our data established the importance of the (−) chiral configuration of DDR for growth suppression of human and dog osteosarcoma cells. We also demonstrated that the C1, but not C5, position of DDR may be modified without significantly affecting its antiproliferative activity. The ability of this class of translation inhibitors, which simultaneously diminishes multiple key mitogenic molecules and induces DNA damage response, G_2_/M arrest, and apoptosis, warrants further consideration as potential therapies for cancers that lack a defined oncogenic driver, such as osteosarcoma.

## Methods

### Compounds.

Isolation of (−)-DDR, (−)-Roc, and (−)-methyl rocaglate from the tropical *Aglaia* plants as part of a multi-institutional collaborative project on the discovery of new antineoplastic natural compounds was as previously described^11,45^. (±)-DDR was synthesized from (±)-methyl rocaglate and separated into its (+)- and (−)-enantiomers on a ChiralPak IB3 and Diacel Chiralcel OD-H columns (Supplementary Figure S1A). Bromo-DDR was generated from (±)-methyl rocaglate, and (±)-DDR-acetate was derived by acetylating (±)-DDR (Supplementary Figure S1B). The details on chemical synthesis and purification of compounds and the verification of their chemical structures and absolute configurations by nuclear magnetic resonance spectroscopy and mass spectrometry are provided in Supplementary Methods. Rhosin, a small molecule inhibitor targeting the RhoA subfamily of Rho–guanosine triphosphatases (GTPases)^25^, was purchased from MilliporeSigma. For cell culture studies, all compounds were prepared as 10 mM (rocaglates) or 25 mM (Rhosin) stock solutions in DMSO and stored at −20°C. For animal studies and some *in vitro* work, (−)-Roc (NSC326408) and (−)-DDR (NSC705956) were chemically synthesized and provided by the NCI Experimental Therapeutics (NExT) Program.

### Cell lines, cell proliferation assay, and flow cytometry.

Human osteosarcoma cells MG-63, OS17, Saos2, and 143B, human Ewing sarcoma cells TC32 and A673, and canine osteosarcoma cells Abrams, OSA8, and OSA16 were previously described^11,46^. Human bone marrow-derived mesenchymal stem cells (MSCs) were kindly provided by Nilay Shah of Nationwide Children’s Hospital. Also, we generated the K9-OS6 cell line from an OSU-K9-OS6 dog osteosarcoma PDX tumor by serial passaging. All cells were grown in Dulbecco’s Modified Eagle’s medium (MilliporeSigma) supplemented with 10% fetal bovine serum (R&D Systems). For single-drug dose-response analysis, osteosarcoma cells seeded in 96-well plates (Sarstedt) were treated for 3 days with compounds added as 9-point, 2-fold serial dilutions. Percent cell viability was assessed by adding resazurin and measuring metabolic conversion to fluorescent resorufin, then averaging fluorescence values of drug-treated wells and normalizing to the DMSO controls set as 100%. Dose-response curves were plotted on GraphPad Prism v9 and the mean absolute 50% growth-inhibitory concentration (IC_50_) values were estimated. For drug combination testing, osteosarcoma cells seeded in 96-well plates were treated for 3 days with (−)-Roc or (−)-DDR arrayed in combination with Rhosin. Viability was assessed by resazurin assays and Bliss synergy scores for each combination calculated using the SynergyFinder v3.0 web-based application (https://synergyfinder.fimm.fi/). For cell cycle analysis, propidium iodide-labeled cells were run on a LSR II flow cytometer (BD Biosciences), followed by gating single cells^47^. The cell cycle distributions were calculated using FlowJo v10 (TreeStar). Cell cycle histograms were deconvoluted using the Dean-Jett-Fox algorithm, and the percentages of cell populations in sub-G_1_, G_1_, S, and G_2_/M calculated.

### Incucyte^®^ live cell imaging and apoptosis assays.

K9-OS6 dog osteosarcoma cells seeded in 96-well plates were treated in duplicate with 2x IC_50_ of (−)-Roc, (−)-DDR, or (±)-DDR-acetate in the presence of Incucyte^®^ Caspase-3/7 Green Apoptosis Assay Reagent (#4440, Sartorius). Percent confluency served as a proliferative readout and green-fluorescence staining counts were used to measure apoptosis (Supplementary Methods). Data were plotted in GraphPad Prism as the mean±SEM of replicate wells.

### Lentiviral-mediated knockdown by short-hairpin RNA (shRNA).

Osteosarcoma cells were seeded at 9,000 cells per well in 6-well plates. The next day, cells in duplicate wells were transduced with 10 multiplicities of infection (MOI) of lentiviruses expressing shRNAs targeting eIF4A1 (TRCN0000288729), eIF4A2 (TRCN0000051869), or both eIF4A1 and eIF4A2 in fresh growth medium containing 8 μg/mL of polybrene (MilliporeSigma). Lentiviruses expressing a nontargeting shRNA sequence (SHC002V) were used as a negative control. Transduced cells were selected with puromycin (2 Mg/mL), and when cells expressing the nontargeting shRNA reached confluence, all wells in the experiment were trypsinized and counted using a hemocytometer. Relative cell numbers were calculated as a percentage of cells transduced with nontargeting shRNA normalized to 100%. Then, cell lysates were prepared from transduced wells and analyzed for protein expression of the eIF4F components (see below).

### Western blotting.

Subconfluent human and dog osteosarcoma cells were treated with 1x or 2x IC_50_ of (−)-Roc, (−)-DDR, (±)-DDR, or (±)-DDR-acetate or 0.04% DMSO vehicle for the indicated times and lysed. Equal amounts of protein lysates were analyzed by Western blotting (see Supplementary Methods and Supplementary Table S4 for detailed procedures and antibodies used).

### Canine PDX model, IHC, and TUNEL staining.

All animal work was performed according to the protocols approved by the Institutional Animal Care and Use Committee at Nationwide Children’s Hospital. The study is reported in accordance with ARRIVE guidelines. Briefly, OSU-K9-OS6 dog osteosarcoma PDX tumor fragments were implanted subcutaneously into the dorsal right flank of eight-to-12-week-old immunodeficient NSG mice *(NOD.Cg-Prkdc*^*scid*^ *Il2rg*^*tmlWjl*^*/SzJ* The Jackson Laboratory) under isoflurane anesthesia as previously described^11^. Tumor measurements were performed using a Fowler Ultra-Cal Electronic Caliper in the axial plane at its greatest dimension (L) and the orthogonal short dimension (S). Mice with actively growing tumors reaching ~ 100–200 mm^3^, calculated using the formula V = (L × S^2^)/2, were randomized into three treatment groups (n = 10/group) and treated with (−)-DDR or (−)-Roc formulated in 30% hydroxypropyl-β-cyclodextrin (HPβCD), or vehicle by intraperitoneal injection (IP) every other day. Lead-in dosing was used with 1 mg/kg of (−)-DDR or (−)-Roc for the first dose, 1.5 mg/kg for the second dose, 2 mg/kg for the third dose, 2.5 mg/kg for the fourth dose, and then the full-dose of 3 mg/kg thereafter. Treated tumors were measured bi-weekly. Following the last measurement, tumors were harvested, fixed in 10% neutral-buffered formalin, and paraffin embedded. Five-micron tumor sections were prepared, deparaffinized, and stained with hematoxylin and eosin (H&E) or immunostained for PCNA or phospho-p38 (p-p38). Apoptosis was assessed by TUNEL staining of tumor sections using the TMR Red *In Situ* Cell Death Detection Kit (Cat. #12156792910; Roche), with DAPI counterstaining to visualize nuclei.

### RNA-seq analysis.

Actively-growing MG-63 and Saos2 cells were treated with 1x IC_50_ of (−)-Roc or (−)-DDR, or DMSO vehicle for 24 hours. Treated cells were harvested on ice, rinsed twice with cold PBS, and centrifuged. The cell pellets were flash frozen at −80°C and submitted to MedGenome, Inc. (Foster City, CA) for RNA-seq analysis. Briefly, RNA was isolated using the Maxwell^®^ RSC simplyRNA Cells kit (Cat. #AS1390; Promega). Libraries were prepared with the Illumina TruSeq stranded mRNA kit and sequenced on an Illumina NovaSeq to generate 100-bp paired-end reads. Before alignment, non-polyA-tailed RNA species, including mitochondrial transcripts, rRNAs, and tRNAs, were filtered out using Bowtie 2 (v.2.5.1)^48^. STAR v2.7.3a^49^ was used to align reads to the GRCh37/hg19 reference human genome (released February 2009). Raw read counts were estimated via HTSeq v0.11.2^50^ and normalized with the DESeq2 package^51^. Cufflinks v2.2.1 was used to estimate the gene expression levels^52^, and the values were reported as FPKM (fragments per kilobase per million) units per gene. DESeq2 was employed to estimate the differential expression of gene counts relative to the DMSO-treated controls. The differentially-expressed genes (DEGs) were filtered, and significant DEGs were defined as transcripts with absolute log2 fold-change ≥ 0.5 and adjusted p-values (Padj) ≤ 0.05. Venn diagrams of significant DEGs were generated using the DeepVenn beta application (http://deepvenn.com/), and volcano plots created using the Galaxy Project web-based bioinformatics platform (http://usegalaxy.org). Gene set enrichment analysis (GSEA) was performed using the GSEAPreranked module of the GenePattern online tool (https://cloud.genepattern.org/). The expression datasets were filtered to remove poorly expressed genes and ranked using the formula Rank = sign(log2FC)*-log10(P-value), with log_2_ fold-change values relative to the DMSO control. Preranked datasets were analyzed using the Hallmark collection of the Molecular Signatures Database^53^.

## Data Availability

RNA-seq data were deposited into the Gene Expression Omnibus database under accession number GSE267810 and are available at the following URL: https://www.ncbi.nlm.nih.gov/geo/query/acc.cgi?acc=GSE267810.
